# Marine Microbiome as a Source of Antimalarials

**DOI:** 10.3390/tropicalmed4030103

**Published:** 2019-07-13

**Authors:** Peter J. McCarthy, Bracken F. Roberts, Abigail Carbonell, Jill Roberts, Amy E. Wright, Debopam Chakrabarti

**Affiliations:** 1 Harbor Branch Oceanographic Institute, Florida Atlantic University, Fort Pierce, FL 34946, USA; 2 Division of Molecular Microbiology, Burnett School of Biomedical Sciences, University of Central Florida, Orlando, FL 32826, USA

**Keywords:** Marine Microbe, Malaria, Plasmodium, Antimalarials, Harbor Branch

## Abstract

It is important to discover novel antimalarial pharmacophores because of the widespread emergence of *Plasmodium falciparum* isolates resistant to the available drugs. Secondary metabolites derived from microbes associated with marine invertebrates are a valuable resource for the discovery of novel drug leads. However, the potential of marine microbes as a source of antimalarials has not been explored. We investigated the promise of marine microorganisms for the production of antimalarial activities by testing 2365 diverse microbial extracts using phenotypic screening of a multidrug resistant chloroquine resistant *P. falciparum* strain. We conducted counter screening against mammalian cells for the 317 active extracts that exhibited more than 70% inhibition at 1 µg/mL. The screen identified 17 potent bioactive leads from a broad range of taxa. Our results establish that the marine microbiome is a rich source of antiplasmodial compounds that warrants in depth exploration.

## 1. Introduction

Over 40% of the global population currently resides in regions with malaria transmission. There were 219 million clinical cases of malaria in 2017 causing 435,000 deaths [1] and the available drugs against malaria are rapidly losing efficacy because of rapid emergence to resistance. Even artemisinin combination therapy, which is the recommended treatment for malaria in the disease endemic countries, is showing an alarming spread of resistance in wide areas of Southeast Asia [2,3]. Given this alarming situation for malaria treatment options, it is important to discover new chemical leads for the next generation of malaria therapeutics.

Historically, natural products have been an important source for the discovery of new drug leads. Of 1562 approved new drugs during the period 1981–2014, 930 (59.5%) were from natural products, natural product-derived, or natural product-inspired compounds [4]. Natural products have been a particularly useful source for the discovery of anticancer and anti-infective agents including antimalarial agents such as quinine. More recently, the artemisinins, derived from the sweet wormwood that was originally used in Chinese herbal medicines, have become a frontline treatment for drug-resistant malaria.

While the majority of the approved natural products and natural product-derived chemical entities are derived from terrestrial habitats, the marine environment remains largely unexplored. This represents an opportunity for the identification of novel compounds with therapeutic properties from marine biodiversity. Although marine invertebrates and algae have been the primary target for the discovery of new therapeutics, the focus of marine biotechnology has now shifted towards marine microbes [5,6]. It is believed that many compounds originally isolated from marine invertebrates actually originate from marine bacteria [5]. The ocean surface layer typically contains 10^5^ microbial cells per ml [7]. Some marine sponges, termed high microbial abundance sponges, contain large populations of associated microbes at densities of 10^8^–10^10^/g sponge tissue that can comprise up to 20–30% of the sponge’s biomass [8] and there is growing evidence that these microbial populations play key roles in sponge metabolism including the production of natural products [9,10]. One of the first examples of a sponge metabolite that has since been shown to be produced by a cultivated microorganism is manzamine A, an antimalarial compound that is produced by a sponge-associated actinomycete [11,12]. Additional antimalarial compounds produced by marine microbes include 2-undecyl-4-quinolone from a sponge-associated *Pseudomonas* [13], and calothrixins A and B from the cyanobacterium *Calothrix* [14]. In spite of the enormous potential of the marine microbiome as a source for novel chemotypes, its utility for discovery of novel antimalarials has not been thoroughly exploited. 

The microbiology group at Harbor Branch Oceanographic Institute (HBOI) has an on-going program studying the microbial population associated with deep-water invertebrates. As part of this research, a focus has been the development of novel cultivation methods that, in certain cases, have improved recovery from environmental samples by over 300% [15]. This ongoing research project has resulted in the establishment of a substantial culture collection of heterotrophic marine microbes, the Harbor Branch Marine Microorganism Culture Collection (HBMMCC, Table 1), which now contains over 19,000 isolates that have only recently become the subject of a focused screening program. We have determined the taxonomic affiliation of >2290 heterotrophic eubacterial isolates in the HBMMCC using restriction fragment linked polymorphism (RFLP) and sequence analyses of the 16S SSU rRNA gene [16,17]. The subset of the HBMMCC that was used for the taxonomic study contains 233 different operational taxonomic units (OTUs) from six major eubacterial clades. SSU rRNA sequences of 19 OTUs were ≤93% similar to the closest sequence match in the GenBank database, indicating the likely discovery of novel microbial taxa. Furthermore, the HBMMCC contains microbes previously described as uncultured, including sponge-specific symbionts. The surveyed actinomycetes indicated that most of these isolates represent new species when compared with those found in GenBank (similarity <97%). The HBMMCC also includes over 2000 fungal isolates including some genera commonly found in many environments, nonetheless, the collection includes more unusual isolates such as *Warcupiella* and a fungus isolated from the sponge *Forcepia* with the closest GenBank match having only 91% sequence similarity to *Penicillium* sp. It is well-documented that marine isolates of common fungi, such as those of the genera *Penicillium* and *Aspergillus*, are known to produce novel metabolites [9,18].

Here we report the screening of the Harbor Branch Marine Microorganism collection for antiplasmodial activities.

## 2. Materials and Methods

### 2.1. Fermentation

Microbial isolates were cultured in SYZ medium (15 g soluble starch, 2 g yeast extract, 4 g NZ-amine, 2 g dextrose, 750 mL artificial sea water, 250 mL deionized water) and KP medium (2 g kelp powder, 2 g fish meal, 4 mL super fish emulsion, 5 g soluble starch, 1000 mL artificial sea water; 30 mL dispensed into flasks containing 60 mg chitosan) using the following two growth conditions. Fermentations were performed primarily in shake cultures (210 rpm, 7–14 days, 25 °C), however a small subset of the isolates were also grown in static cultures (~21 days, 25 °C). All liquid fermentations were performed at the 30 mL scale using 125 mL Erlenmeyer flasks. 

An alternate fermentation method used moistened rice as a substrate for the growth of fungi. Rice (25 g) was placed in a 125 mL flask and soaked overnight in 100 mL deionized water. The water was then decanted off and the flask capped. Autoclaved flasks were inoculated from a 10 mL SYZ liquid culture and incubated at 25 °C for ~21 days. At harvest, the cultures were freeze-dried prior to extraction.

### 2.2. Extraction

At harvest, a 1:1 mixture of Amberlite^®^ XAD-16 (Sigma-Aldrich, St Louis, MO, USA) and Diaion^®^ HP20 (Sigma-Aldrich, St Louis, MO) resins was added to liquid cultures and mixed for 2 h on a rotary shaker. Resins were added in order to extract a broad range of metabolites from the fermentation broth. The mixture of cell mass and resins were collected by filtration through Celite^®^ 545 (Acros Organics, Thermo Fisher Scientific, Waltham, MA, USA). The cell residue and resin was washed 3× with 50 mL deionized water, to remove broth components and salts, and was then extracted sequentially by the addition of methanol (50 mL) and dichloromethane (50 mL). 

Extraction of moistened rice fermentations was performed using an Accelerated Solvent Extractor (ASE 100, Dionex, Salt Lake City, UT, USA). Freeze-dried samples were packed into 100 mL extraction cells and extracted at 100 °C by the sequential addition of heptane, ethyl acetate:ethanol (1:1), and methanol. Each solvent was added twice with a soak time for each addition of 21 min. The extraction system was designed to extract chemicals with a broad polarity range. 

All extracts were concentrated under reduced pressure and subsamples (200 µg) of each were submitted for biological evaluation. 

### 2.3. Plasmodium in Vitro Culture

*Plasmodium falciparum* chloroquine resistant Dd2 was maintained in a modified Trager and Jensen method [19] in RPMI 1640 medium with l-glutamine (Invitrogen, Carlsbad, CA, USA) and supplemented with 25 mM HEPES, pH 7.4, 26 mM NaHCO_3_, 2% dextrose, 15 mg/L hypoxanthine, 25 mg/L gentamycin, and 0.5% Albumax I in human A^+^ erythrocytes. Cultures were incubated at 37 °C in a humidified environment of 5% CO_2_ and 95% air.

### 2.4. Antiplasmodium Activity Assay

A SyBR green I-based fluorescent assay which measures the DNA content of the parasite was used to measure inhibitory property of extracts [20,21,22]. The dried extracts were reconstituted in 100% DMSO or ethanol, and were serially diluted in culture medium as needed and added to the parasite culture (Dd2 line) at 1% parasitemia and 1% hematocrit in 96-well black plates (Greiner Bio-One, Monroe, NC, USA). The final concentration of the vehicles in the assay did not exceed 0.2%, which was used as a negative control. Following incubation for 72 h at 37 °C the plates were frozen at −80 °C and thawed. Lysis buffer (20 mM Tris-HCl, 0.08% saponin, 5 mM EDTA, 0.8% Triton X-100, and 0.01% SYBR Green I) was added to each well and incubated in the dark for 30 min at 37 °C. Fluorescence emission from the wells was measured using a Synergy H4 multimode plate reader (Biotek, Winooski, VT, USA) at wavelengths 485 nM for excitation and 530 nM for emission.

### 2.5. Cytotoxicity Assay

Dilutions of extracts were evaluated for cytotoxicity [23] in mouse 3T3 fibroblast cells (2500 cells/well) in 384 well clear bottom plates (Santa Cruz Biotechnology, Dallas, TX, USA). The plates were incubated for 48 h at 37 °C in a humidified environment (5% CO_2_, 95% air). Following the incubation period 20 µL MTS ((3-(4,5-dimethylthiazol-2-yl)-5-(3-carboxymethoxyphenyl)-2-(4-sulfophenyl)-2*H*-tetrazolium), CellTiter 96^®^ Aqueous nonradioactive cell proliferation assay, Promega) reagent was added to each well, the plates were further incubated for an additional 3 h. Cell viability data were obtained by measuring absorbance at 490 nm using Synergy H4 plate reader (Biotek, Winooski, VT, USA).

### 2.6. Dereplication of Bioactive Fractions

V324, KJ1233. The methanol extract (151 mg) from V324 (KJ1233) was chromatographed using medium performance liquid chromatography (MPLC) using a Combiflash™ RFx4 flash chromatography instrument (Teledyne Isco, Lincoln NE). The sample was bound to C-18 packing, packed in a loading cartridge and then chromatographed on a 4.3 g RediSep Gold C-18 column using a gradient from H_2_O:CH_3_CN (9:1) to 100% acetonitrile over 32 column volumes (CV), followed by isocratic CH_3_CN for 10 CV then washed with methanol for 4 CV and then a methanol to 100% dichloromethane wash over 12 CV. The flow rate was 18 mL/min. The resulting material was collected into 44 tubes, which were then combined into 12 fractions based upon observed peaks at 220 nm and 254 nm. The fractions were dried down through distillation under reduced pressure, transferred into pre-weighed vials, and brought up into 10 mg/mL solutions for analysis and subsampling for bioassay. 

V881, KJ1242. The methanol extract (515 mg) from V881, (KJ1242) was chromatographed using medium performance liquid chromatography (MPLC) using a Combiflash™ RFx4 flash chromatography instrument (Teledyne Isco, Lincoln, NE). The sample was bound to C-18 packing, packed in a loading cartridge and then chromatographed on a 13 g RediSep Gold C-18 column using a gradient from H_2_O:CH_3_CN (95:5) to 100% acetonitrile over 20 column volumes (CV), followed by isocratic CH_3_CN for 5 CV then washed with methanol for 2.5 CV and then a methanol to 100% dichloromethane wash over 6 CV. The flow rate was 30 mL/min. The resulting material was collected into 71 tubes, which were then combined into 10 fractions based upon observed peaks at 220 nm and 280 nm. The fractions were dried down through distillation under reduced pressure, transferred into pre-weighed vials, and brought up into 10 mg/mL solutions for analysis and subsampling for bioassay. 

## 3. Results

### 3.1. Extracts of Marine Microbes Used in Screening

A library of 2365 marine microbial extracts was prepared from the HBMMCC. These were derived from 694 isolates from the HBMMCC: 316 fungi (F), 45 actinomycetes (A) and 332 other bacteria (157 gram-negative (B (G-)) and 175 gram-positive B (G+)). For this work we used a variety of fermentation conditions: 46.6% of the extracts were from SYZ shake cultures; 25.8% from KP shake cultures; 26.0% from moistened rice cultures; 0.8% from static SYZ cultures; and 0.8% from static KP cultures. The average extract yield was 130 mg (range 50--300 mg) and provided sufficient material for both screening and preliminary chemical studies.

### 3.2. Screening of HBMCC Extracts for Antiplasmodial Activities

**Figure 1:**
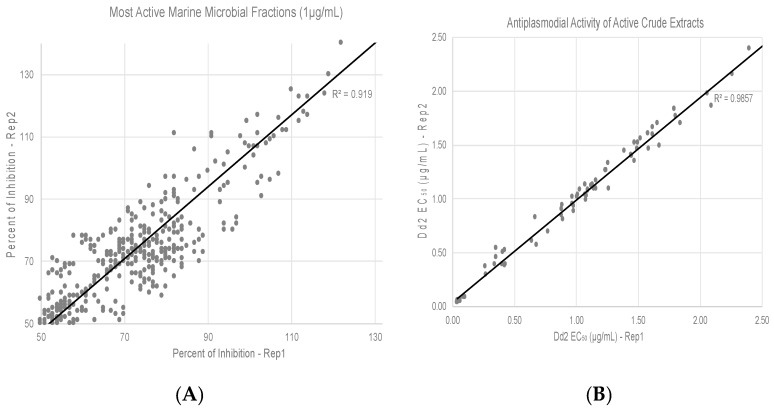
Antiplasmodial activities of marine microbial extracts. (**A**) Active extracts with ≥50% inhibition at 1 µg/mL. Percent inhibition of *P. falciparum* Dd2 growth in two different replicates (Rep1 and Rep2) of SYBR green-I fluorescence assay is shown in a correlation plot. (**B**) Fifty percent inhibitory concentrations (EC50) of top hit extracts are shown.

We used SYBR green I-based fluorescence assay which measures inhibition of growth in terms of reduction in DNA content to measure antiplasmodial activities of marine microbial extracts. Initial screening of 2365 diverse marine microbial extracts was carried out at a concentration of 10 µg/mL using the chloroquine-resistant Dd2 strains under standard culture conditions [24] for 72 h. For our primary screen, we used the chloroquine-resistant Dd2 strain as our main goal is to overcome the problem of drug-resistant malaria. All plates contained control wells: (a) culture only and (b) with chloroquine. Z’ factor, which is a statistical parameter of assay quality [25] was calculated and only those assays with values >0.7 were considered for evaluation. An extract was considered as a potential “hit” when it exhibited at least 70% inhibition of growth at 10 μg/mL. Extracts exhibiting ≥ 70% inhibition were subjected to screening at 1 µg/mL; Figure 1A shows the active extracts with ≥50% inhibition at 1 µg/mL. The extracts active ≤ 1 µg/mL were subjected to EC_50_ determination (Figure 1B).

We also determined the cytotoxicity of active fractions against NIH 3T3 fibroblasts using an MTS cell proliferation assay [23]. As can be seen from Table 2, 17 fractions from diverse microbial sources exhibited promising activities with EC_50_ values ≤ 1 µg/mL It is to be noted that active fractions also have good selectivity of >10.

### 3.3. Identification of Bioactive Compounds

Isolates V324 and V881. The first sample that was investigated was the methanol extract from V324, extract ID: KJ1233. MPLC fractionation yielded three active fractions, which upon interpretation of the NMR spectra suggested that they appear to be mixtures of phosphatidyl choline analogs. Full structure characterization was not conducted, as this class of compound is known to show strong antiplasmodial activity [26]. 

The second sample that was investigated was organism V881, extract ID: KJ1242. The active extract exhibited a promising EC_50_ of 0.062 µg/mL. MPLC fractionation yielded ten fractions. The ^1^H NMR spectra of the most potent fractions were complex with large resonances attributable to lipids and other very minor components with aromatic, olefinic and hetero substituted methine resonances. The LC-MS signal was also complex showing the presence of multiple compounds. The fraction was a light purple in color and a number of HPLC separations were attempted on different C-18 and amino substituted stationary phases. In all cases it appeared that the purple compound was associated with bioactivity but it was present in very low amounts. This compound also showed unusual chromatography in that at times it appeared quite polar and at other times less polar. Unfortunately, the active compound was never fully purified, and its structure remains to be identified. Further work including large scale up of the culture is required to fully identify the structure of the compound. 

## 4. Discussion

This project screened a subset of the Harbor Branch Marine Microbial Culture Collection for antiplasmodial activity. Of the 2365 extracts tested, 317 (13%) exhibited 70% inhibition when tested at 1 µg/mL (Figure 1). From these preliminary hits we were able to identify 17 extracts which could be considered leads based on their inhibitory activity against the chloroquine-resistant *P. falciparum* and their cytotoxicity towards mammalian cells. The leads were produced from actinomycetes, fungi and gram-negative bacteria. Interestingly, no non-actinomycete gram-positive bacteria were found to be leads even though they comprised over 25% of the isolates tested. The leads came from both KP and SYZ liquid fermentations with no overlap between the two media even though many isolates were grown on both media. It is most interesting that 5/17 leads were generated from static liquid cultures even though only 1.6% of the extracts came from this type of fermentation. 

The leads that were generated by this project came from a broad range of taxa. The five fungal isolates were *Penicillium* spp., *Talaromyces rotundus*, which was considered a teleomorph of *Penicillium* but is now considered a distinct phylogenetic lineage [27], and *Tritirachium* sp. There is an extensive literature on the natural products of terrestrial *Penicillium* and other fungi [28]; however, recently, marine isolates from these genera have proven to be the source of novel natural products [18]. The same is true for *Talaromyces* spp., which are common mangrove endophytes and which have been shown to produce a broad range of natural products [18,29]. Marine strains of *Tritirachium* have also received attention for their production of natural products, including xanthoquinodin-like compounds from a *Tritirachium* isolated from a marine sponge [30].

The six strains of actinomycetes that showed activities are also not unexpected as, in general, these gram-positive bacteria are prolific producers of bioactive natural products [31]. It was, however, surprising to find that six isolates of gram-negative bacteria showed activity in our screen. Gram-negative bacteria have recently been recognized as a potentially valuable resource for the discovery of natural products including polyketides, nonribosomal peptides, hybrid polyketide-nonribosomal peptides and others [32]. Three of our active isolates belong to the genus *Marinobacter*, which has been found to produce the siderophore petrobactin [33]. However, other natural products and their biological activity have not been reported. The oil-degrading bacterium *Alcanivorax* is known to produce surfactants [34] as well as the α-pyrone alcanivorone [35] but no biological activity has been reported for this molecule. *Endozoicomonas* spp. are known to be associated with tunicates [36], sponges [37], and octocorals [38], where they can become dominant members of the microbial community. There have been no studies of their secondary metabolites but one paper described antibacterial activity of *Endozoicomonas* extracts [37]. 

Our initial attempt to identify the structure of bioactive component of V881 was inconclusive. We now recognize the interference of commonly encountered compounds such as fatty acids and choline derivatives that will have to be dereplicated early in the discovery process in order to find the more structurally complex natural products with potential for development as pharmaceutical agents.

Our results support the hypothesis that marine microorganisms have a very high likelihood of yielding novel antimalarial chemotypes. From the isolates producing high levels of activity we have found taxonomic diversity, which we expect to translate into chemical diversity with continued study. Based on the taxonomy of the producing organisms we expect to find novel chemotypes from this ongoing project.

## Figures and Tables

**Table 1:** Taxonomic affiliation of isolates contained in the HBOI Marine Microbial Culture Collection.

Taxonomic Affiliation	Number of Isolates	Number Isolated from Sponges
Actinomycetes	1037	416
Other bacteria	16,129	11,281
Fungi	2074	1427

**Table 2:** Activity of promising extracts.

Isolate #	Taxonomy	Category	Medium	Incubation Period	Growth Conditions	Extraction System	Dd2 IC_50_ µg/mL	Cytotoxicity IC_50_ µg/mL
V324	*Streptomyces tendae*	A	SYZ	22 days	Static	Resin, MeOH/CH_2_Cl_2_	0.35	>50
V663	Unidentified actinomycete	A	Rice	21 days	Static	ASE, Heptane	0.89	10.2
V671	*Nocardiopsis* sp.	A	Rice	21 days	Static	ASE, MeOH	0.88	9.1
W305	*Micromonospora* sp.	A	SYZ	14 days	Shake	Resin, MeOH	0.42	9.3
V881	*Streptomyces* sp.	A	SYZ	14days	Shake	Resin, CH_2_Cl_2_	0.062	29.1
Z691	*Penicillium* sp.	F	SYZ	14 days	Shake	Resin, CH_2_Cl_2_	0.049	27.2
E677	*Streptomyces* sp.	A	SYZ	7 days	Shake	Resin, MeOH/CH_2_Cl_2_	0.037	28.6
H402	*Endozoicomonas numazuensis*	B (G-)	KP	7 days	Shake	Resin, MeOH/CH_2_Cl_2_	0.978	>50
N161	*Penicillium* sp.	F	SYZ	22 days	Static	Resin, MeOH/CH_2_Cl_2_	0.266	>50
S920	*Talaromyces rotundus*	F	KP	21 days	Static	Resin, MeOH/CH_2_Cl_2_	0.677	>50
V170	*Penicillium citrinum*	F	KP	21 days	Static	Resin, MeOH/CH_2_Cl_2_	1.069	>50
V174	*Alcanivorax* sp.	B (G-)	KP	7 days	Shake	Resin, MeOH/CH_2_Cl_2_	0.969	>50
V184	*Marinobacter* sp.	B (G-)	KP	7 days	Shake	Resin, MeOH/CH_2_Cl_2_	1.008	>50
V193	*Alcanivorax* sp.	B (G-)	KP	7 days	Shake	Resin, MeOH/CH_2_Cl_2_	1.079	>50
V199	*Tritirachium* sp.	F	KP	7 days	Shake	Resin, MeOH/CH_2_Cl_2_	0.339	>50
V201	*Marinobacter* sp.	B (G-)	SYZ	24 days	Static	Resin, MeOH/CH_2_Cl_2_	1.091	>50
V208	*Marinobacter* sp.	B (G-)	SYZ	7 days	Shake	Resin, MeOH/CH_2_Cl_2_	1.091	>50
